# MEG-BIDS, the brain imaging data structure extended to magnetoencephalography

**DOI:** 10.1038/sdata.2018.110

**Published:** 2018-06-19

**Authors:** Guiomar Niso, Krzysztof J. Gorgolewski, Elizabeth Bock, Teon L. Brooks, Guillaume Flandin, Alexandre Gramfort, Richard N. Henson, Mainak Jas, Vladimir Litvak, Jeremy T. Moreau, Robert Oostenveld, Jan-Mathijs Schoffelen, Francois Tadel, Joseph Wexler, Sylvain Baillet

**Affiliations:** 1 McConnell Brain Imaging Centre, Montreal Neurological Institute, McGill University, Montreal QC, H3A 2B4, Canada; 2 Center for Biomedical Technology, Universidad Politécnica de Madrid, 28223 Madrid, Spain; 3 Department of Psychology, Stanford University, Stanford, CA 94305, USA; 4 The Wellcome Centre for Human Neuroimaging, UCL Institute of Neurology, UK; 5 LTCI, Telecom ParisTech, Université Paris-Saclay, 75013 Paris, France; 6 INRIA, Université Paris-Saclay; 7 MRC Cognition and Brain Sciences Unit, University of Cambridge, Cambridge, CB2 7EF, UK; 8 Donders Institute for Brain, Cognition and Behaviour, Radboud University, 6500 HE Nijmegen, Netherlands; 9 NatMEG, Department of Clinical Neuroscience, Karolinska Institute, SE-171 77 Stockholm, Sweden; 10 Inserm, U1216, F-38000, Grenoble, France; 11 Univ. Grenoble Alpes, Grenoble Institut des Neurosciences, GIN, F-38000 Grenoble, France

**Keywords:** Neuroscience, Electrophysiology, Research data

## Abstract

We present a significant extension of the Brain Imaging Data Structure (BIDS) to support the specific aspects of magnetoencephalography (MEG) data. MEG measures brain activity with millisecond temporal resolution and unique source imaging capabilities. So far, BIDS was a solution to organise magnetic resonance imaging (MRI) data. The nature and acquisition parameters of MRI and MEG data are strongly dissimilar. Although there is no standard data format for MEG, we propose MEG-BIDS as a principled solution to store, organise, process and share the multidimensional data volumes produced by the modality. The standard also includes well-defined metadata, to facilitate future data harmonisation and sharing efforts. This responds to unmet needs from the multimodal neuroimaging community and paves the way to further integration of other techniques in electrophysiology. MEG-BIDS builds on MRI-BIDS, extending BIDS to a multimodal data structure. We feature several data-analytics software that have adopted MEG-BIDS, and a diverse sample of open MEG-BIDS data resources available to everyone.

## Introduction

The Brain Imaging Data Structure (BIDS) is an emerging standard for the organisation of neuroimaging data (https://bids.neuroimaging.io)^1^. The significance of BIDS is timely: there is increasing availability of open neuroimaging data resources, and strong interest in aggregating large, heterogeneous datasets to harness a new range of scientific questions, and augment statistical power. This also poses new challenges in terms of data organisation, harmonisation, (re)processing and sharing, especially without a standard format for digital neuroscience data between or within data modalities (e.g., in electrophysiology).

Consequently, present data management practices do not generalise between labs, or even between members of the same research group. This leads to suboptimal usage of human (time lost retrieving data), infrastructure (data storage space) and financial (limited longevity and value of disorganised data after first publication) resources. Poor or lacking data management strategies also negatively affects the reproducibility of results, even within the lab where the data were collected.

BIDS was initiated with magnetic resonance imaging (MRI) data. It provides a simple, hierarchical folder structure, with key study parameters documented in text-based metadata files. MRI-BIDS can manage multiple MRI modalities, with minimal curation overheads. This reduces the likelihood of data-handling errors. Another key benefit is the augmented interoperability between BIDS-compatible software tools for data analytics.

Here we introduce a significant extension of BIDS to electrophysiology data. The technical sophistication of magnetoencephalography (MEG) makes it the most challenging electrophysiology data type for standardisation^2^. MEG-BIDS can therefore readily be generalised to electroencephalography (EEG), multiunit recordings, and local field potentials. Further to strengthening and rationalising data management in MEG labs, MEG-BIDS provides a common structure for present and future large MEG open-data repositories^3–5^. The new standard handles files collected from all types of MEG instruments, regardless of their raw data file format (e.g., CTF/VSM, Elekta/Neuromag/MEGIN, Yokogawa/KIT, Neuroscan/KRISS, etc. and more can be readily added to accommodate the newest MEG manufacturers and as format versions evolve.) The absence of a unique data file format in MEG is indeed compensated by MEG-BIDS’s standard data organisation and metadata principled reporting: the sharing and processing of large and complex data hierarchies is simplified, and made compatible and reproducible across tools for data analytics.

### MEG-BIDS specifications

The MEG-BIDS specifications were defined in accord with best-practice guidelines for conducting MEG research^6^, with direct inputs from MEG investigators, technical support staff and data managers. The teams of leading academic data-analysis software were also directly involved^7^: Brainstorm^8^, FieldTrip^9^, MNE^10^, and SPM^11^, which all have adopted MEG-BIDS. The design process was initiated at the 2016 International Conference on Biomagnetism (Seoul, South Korea), where the first incarnation of MEG-BIDS was introduced, and open group discussions ensued.

A first version of the present manuscript was shared as a preprint^12^, and received comments and suggestions from the community to refine the MEG-BIDS specifications. In July 2017, we published an open poll survey for the MEG community to express their potential interest in MEG-BIDS. The poll received 78 international entries, with most salient results indicating i) strong interest in a common standard for MEG data organisation (98.7%), ii) affirmed willingness to try a MEG-BIDS solution (97.4%).

MEG-BIDS shares common data descriptors with BIDS, such as *Subject*, *Session, Technique,* and *Run*. In MEG terminology, *Subject* refers to a scanned participant. We acknowledge that MEG is not strictly a *scanning* technique. Yet, this is for convenience, affinity with other neuroimaging modalities, and to reflect the language used in most MEG labs. ‘*Session*’ defines a non-intermittent period of time during which the subject is in the scanner. ‘*Run*’ is a period of time during which brain activity or empty-room (for noise characterisation) is recorded continuously, with no interruptions. It is typical with MEG that a *Session* consists of multiple *Runs*: task instructions can change and/or participants can take a break between runs. The notion of *Task* refers to the instructions (and corresponding stimulus material) that are performed by the participant. *Responses* is a feature for continuous recording of behaviour. Note that BIDS also supports storage of stimuli files (such as videos), and annotations of events via a hierarchical event descriptor extension. MEG-specific metadata elements can be reported with the widely used JavaScript Object Notation (JSON) and Tab Separated Value (TSV) text file formats, both human- and machine-readable, which contributed to the versatility and practicality of MRI-BIDS.

The comprehensive MEG-BIDS specifications are openly available elsewhere^13^, and include links to main BIDS specifications and JSON schemas. Note that there are no JSON LD context files, but with attention to backward compatibility with present BIDS specifications, they could be added in future versions. There is also ongoing work in the BIDS community to reach to the NIDM (Neuroimaging Data Model) efforts to expose experiment metadata as linked data. In the same vein, with MEG-BIDS extending from original MRI-BIDS, it does not support Resources Description Format (RDF) representations. The original decision for BIDS to not rely on RDF was driven by the complexity of that format and relatively low familiarity among scientists and software developers outside of the semantic web circles. Yet, current investigations of BIDS to NIDM conversions may lead to RDF views of BIDS datasets.

The MEG-BIDS file organisation also extends, with full compatibility, that of MRI-BIDS: file names are constituted by a series of key-value pairs, with multiple possible file types, while retaining a few typological aspects. MEG-BIDS can therefore register data of any kind, including but not limited to task-based, resting-state, and empty-room data collection. We emphasise that all of the above notions apply also to EEG and all other modalities of electrophysiology, for which MEG-BIDS serves as template for standardisation.

Such flexibility was crucial to retain for MEG, as we already mentioned there is no common, open or standard file format in the field. MEG-BIDS stores unprocessed (raw) data in the native vendor file format. This means that users can still rely on their preferred data review application, possibly provided by the system’s vendor, for files stored in the MEG-BIDS folder structure. Free, open-source BIDS-compliant software (see below) can also extract most required meta information elements from these raw data files, such as data collection parameters and other study and task descriptors. They are eventually transcribed into sidecar JSON files by these MEG-BIDS compatible applications. One major benefit of metadata extraction is the facilitation of subsequent data searches and indexation, without the handling and repeated parsing of large raw data files. Additional relevant files can be included alongside the MEG raw data: some propositions are detailed in the online specifications^13^.

**Figure 1:**
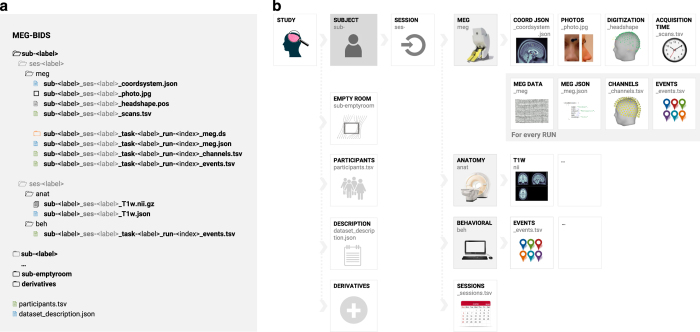
Overview of MEG-BIDS data organisation. (**a**) MEG-BIDS data organisation scheme. MEG-BIDS organises data per study, then participant (subject), followed by sessions, modality and eventually, runs. Note the sidecar files present at all levels of the data hierarchy, conveniently documenting the metadata contents. Further organisational details are illustrated in (**b**) MEG-BIDS general overview, featuring some the expected types of contents and information pertaining to the study, participant, and related multimodal data.

MEG-BIDS describes a hierarchical file structure that descends from a *Study* folder. Multiple *Subject* subfolders contain the data from the participants enrolled. They are arranged by *Session*, each session subfolder containing *Run* folders and eventually, data and metadata files (Fig. 1).

The *Run* folder includes a variety of files: MEG recording files in native format, a sidecar JSON document (*_meg.json), a channel description table (*_channels.tsv), and other general BIDS files, such as task events tables (*_events.tsv) that are likely to be specific of each run. *Session* specific files include the coordinates of anatomical landmarks and head-localisation coils stored in a JSON document (*_coordsystem.json) together with the fiducials information, optional photographs of the anatomical landmarks and/or head localisation coils (*_photo.jpg), 3-D scalp digitalisation files (*_headshape.<manufacturer_specific_format>) and acquisition times (scans.tsv). The *Subject* and *Study* specific files are inherited directly from the general BIDS specifications (e.g., participants.tsv). Note that in case of conflict between fields of different runs/sessions, the inheritance principle should be applied: the description file closer to the data prevails^1^.

One issue that required special attention was the multiplicity of coordinate systems and units between MEG systems. To impose a unique coordinate system for BIDS based on the subjects’ brain anatomy (e.g., MNI coordinates or equivalent) was an appealing solution, which however would lack generalizability in the MEG practice. For instance, MEG data can be collected without anatomical information, such as empty-room noise recordings, which are important to optimize source modeling^2^. MEG-BIDS therefore associates all recordings with anatomical fiducial locations stored in a dedicated field of the MRI json file. Again, MEG-BIDS compatible software can read and interpret this information properly.

The BIDS online validator was updated to accommodate its MEG extension (with open code available at GitHub: https://github.com/INCF/bids-validator and the actual online tool at: https://incf.github.io/bids-validator/). It is available for users to verify the validity of their MEG-BIDS data preparation.

### Free, open MEG-BIDS data samples

We provide four publicly-available data repositories in MEG-BIDS format, for a total of 200GB. They are freely accessible for download in the public domain via OpenNeuro. Note that for educational and demonstration purposes, the International Neuroinformatics Coordinating Facility’s GitHub also hosts a lighter version (data structure only, no actual MEG data provided) of the MEG-BIDS dataset examples (https://github.com/INCF/BIDS-examples).

1. **Resting-state**^14^ (https://openneuro.org/datasets/ds000247): Five minutes of eyes-open, resting-state MEG data (5 subjects from OMEGA^4^, with individual T1 MRI; 10.26GB). This dataset is described in Brainstorm’s specific MEG-BIDS tutorial (http://neuroimage.usc.edu/brainstorm/Tutorials/RestingOmega).
2. **Auditory task**^15^ (https://openneuro.org/datasets/ds000246): MEG-BIDS version of an auditory-stimulation study (1 subject with T1 MRI; 2.3GB). The data is described in detail in the main online tutorial of Brainstorm (https://neuroimage.usc.edu/brainstorm/Tutorials).
3. **Visual and auditory tasks**^16^ (https://openneuro.org/datasets/ds000248): MNE data sample (1 subject; 185.85MB) from a previously published study^10^ (includes T1 and FLASH MRI). This dataset is described in detail in MNE’s online tutorial (https://martinos.org/mne/stable/manual/sample_dataset.html).
4. **Visual group study**^17^ (https://openneuro.org/datasets/ds000117): A multi-modal (MEG, EEG, fMRI) human neuroimaging dataset of 16 subjects from a previously published study on face processing^18^ (60.88GB): This dataset is described in detail in the SPM12 manual (www.fil.ion.ucl.ac.uk/spm/doc/manual.pdf).

### Open-source and publicly-available MEG-BIDS compatible software and tools

Widely-used free and open MEG (also for EEG and electrophysiology) software packages have already added functionality to support MEG-BIDS. All feature basic preprocessing tools and advanced solutions for sophisticated data analytics e.g., source imaging, time-frequency decompositions, statistical inference, multivariate decoding, and measures of functional connectivity:

1. **Brainstorm**^8^: Matlab and Java application, with rich graphical-user interactions and analytic pipeline designs for MEG, EEG, NIRS, and electrophysiology recordings. BIDS-formatted MEG/EEG datasets can be imported automatically into the Brainstorm database, as described in the above-mentioned MEG-BIDS Resting-state tutorial using the OMEGA sample.
2. **FieldTrip**^9^: Matlab toolbox for MEG, EEG, and other electrophysiological data full analysis pipelines. Among others, FieldTrip has been used for the MEG part of the Human Connectome Project.
3. **MNE**^10^: a Python software package with MEG analysis tools and workflows. MNE provides code to read and write files in MEG-BIDS-compatible format. Eventually, the MNE team has committed to distribute all its tutorial datasets in MEG-BIDS, along with and the relative analysis scripts. A preliminary version is available at https://mne-tools.github.io/mne-bids/.
4. **SPM**^11^: software written in Matlab, where many widely-used methods for the analysis of PET, fMRI and for computational neuroanatomy were originally developed and implemented. SPM has been extended to MEG and EEG analyses and SPM12 includes the *spm_BIDS* library to parse and query BIDS-formatted datasets, as well as low-level functions to read/write JSON and TSV metadata files. A complete pipeline for the analysis of a group MEG-BIDS dataset is also available.
5. **MEG-BIDS JSON file generator:** Python scripts to produce the JSON sidecar files and reduce the overhead of adopting MEG-BIDS: publicly-available (https://github.com/INCF/pybids).
6. **MEG-BIDS validator (http://incf.github.io/bids-validator/) :** Developed in JavaScript using Node.js, it can be packaged to work in the browser (Google Chrome). A command line version is also provided so that it can be used in scripted analysis. The validator performs several sanity checks on datasets with ensure they are compatible with BIDS. This includes the use of regular expressions to check filenames, and JSON schemas to ensure that the metadata files are standardised by data types.

## Discussion

MEG-BIDS is a proposal to establish a standard framework for the organisation of electrophysiology data. It does not impose the standardisation of the data file format *per se*. Most present and future tools for MEG data analytics can be readily equipped with the necessary readers for all existing vendor formats: these libraries are available in open source from the above-cited software. We believe the capacity of MEG-BIDS to organise data without requiring a common data format is actually a strength: the standard is flexible enough for any data parameters to be extracted and stored as metadata in sidecar JSON files at the time of creating a new MEG-BIDS data entry, regardless of the original file format of the raw data. Therefore, any new data format for electrophysiology is by design compatible with MEG-BIDS^19^.

Along the same lines, MEG-BIDS handles the different coordinate systems defined by MEG vendors, provided that they are documented in the *_coordsystem.json file. This extends to the coordinate systems used for MEG and EEG sensor locations, MRI volumes, anatomical fiducials, landmarks and digitised head points.

Following this seminal contribution, we will extend MEG-BIDS towards the handling of processed data, which for now and akin to MRI-BIDS, are simply stored in a data *derivatives* folder.

We anticipate that the systematic data organisation enabled by MEG-BIDS will be supported by an increasing number of neuroimaging tools, and that more publicly-available data repositories will adopt the standard. The straightforward design of MEG-BIDS makes it an interoperable common exchange format for transferring data between investigators and community repositories e.g., OMEGA^4^ and OpenNeuro^20^. It also facilitates multimodal integration (between MRI, fMRI, MEG, etc.), as the data from multiple modalities can follow the same organisation scheme.

## Additional information

**How to cite this article**: Niso, G. *et al*. MEG-BIDS, the brain imaging data structure extended to magnetoencephalography. *Sci. Data* 5:180110 doi: 10.1038/sdata.2018.110 (2018).

**Publisher’s note**: Springer Nature remains neutral with regard to jurisdictional claims in published maps and institutional affiliations.
